# Arnold‐Chiari malformation type 1 as an unusual cause of acute respiratory failure: A case report

**DOI:** 10.1002/ccr3.3043

**Published:** 2020-06-17

**Authors:** Mohamad Y. Khatib, Moustafa S. Elshafei, Amr M. Shabana, Dnyaneshwar P. Mutkule, Dinesh Chengamaraju, Nevin Kannappilly, Nazeer Alaudeen, Ruchir Milind Joshi, Abdulqadir J. Naswhan

**Affiliations:** ^1^ Hazm Mebaireek General Hospital (HMGH) Hamad Medical Corporation (HMC) Doha Qatar; ^2^ Medical Intensive Care Department Hazm Mebaireek General Hospital (HMGH) Hamad Medical Corporation (HMC) Doha Qatar; ^3^ University of Calgary in Qatar (UCQ) Doha Qatar

**Keywords:** acute respiratory failure, arnold‐chiari malformation, arnold‐chiari malformation type 1

## Abstract

The authors urge clinicians to consider the possibility of Arnold‐Chiari Malformation type 1 with other central causes especially in cases where acute respiratory failure is unexplainable.

## Graphical abstract

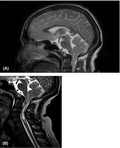

## 1 BACKGROUND

Chiari malformations are a group of heterogeneous disorders; Chiari I malformation is characterized by a form of structural defect in the cerebellum and the cranial base. The case report highlights the possibility of underlying neurological disease in cases where acute type 2 respiratory failure is unexplainable.

Historically, Hans von Chiari described certain hindbrain abnormalities as postmortem findings in infants; these came to be known as Chiari malformations. Four types of Chiari malformations are described in the literature: types I, II, III, and IV.^1^, ^2^ (Chiari malformation types II, III, and IV are distinct from type I and are not discussed in this article). Chiari I malformation is the most common, having been estimated to occur in 1 in 1000 births.^1^

Arnold‐Chiari type I malformation (type I ACM or CM‐I) is characterized by cerebellar tonsils herniation and downwardly displaced below the level of the foramen magnum. Typically, tonsils lying 5 mm or more (normally 3 mm) below the foramen magnum on neuroimaging are consistent with an ACM. However, there is no known direct correlation between clinical severity and the tonsils position.

The actual nature of CM‐I has not been fully understood.^2^ In most cases, CM‐I does not become symptomatic until adolescence or adulthood^3^, ^4^, ^5^ and the mean age at presentation is approximately 18 years.^6^ Also, symptom onset is often insidious. Usually, symptoms of type I ACM develop as a result of the following mechanisms: cerebellum, medulla, and upper spinal cord compression, and disruption of cerebral spinal fluid (CSF) flow through the foramen magnum. Medulla and spinal cord compression may result in spinal cord injury and lower cranial nerve and nuclear dysfunction. Also, the cerebellum compression may result in various neurological symptoms such as dysequilibrium, dysmetria, ataxia, and nystagmus. Disruption of the CSF flow through foramen magnum probably leads to the most common symptom, which is pain.

## 2 CASE PRESENTATION

A 37‐year‐old, healthy, nonsmoker male construction worker with no prior history of comorbidities attended our emergency department with a history of dry cough, fever, and shortness of breath for three days. His respiratory rate was 20/min with O2 saturation of 94% on a nasal cannula 4 L/m, hypotensive on norepinephrine, looked confused but obeyed commands.

Chest radiography showed nonhomogeneous opacities in the right mid‐ and lower zones. Arterial blood gas on admission revealed hypoxemia (PaO2 71 mm Hg) and mild hypercapnia (PaCO2 43 mm Hg), high bicarbonate concentration (23.6 mmol/L) and pH was 7.366 with normal lactate. Initially, he was started on broad‐spectrum antibiotics. Initially, his oxygenation maintained with a nasal cannula. Subsequent arterial blood gases (ABGs) showed elevation PaCO_2_ and was supported with bilevel positive airway pressure ventilation (Bi‐PAP) alternated with high flow nasal cannula. The respiratory viral panel came positive for rhinovirus polymerase chain reaction (PCR) and human metapneumovirus PCR, and septic workup was negative.

He improved clinically and weaned from norepinephrine for over 24 hours. However, he continued to require BiPAP due to type 2 respiratory failure with noted episodes of bradypnea (8‐10 BPM) and dropped oxygen saturation during sleep. Drug overdose was ruled out, and thyroid functions were normal. He developed mild occasional difficulty of swallowing on 6th day of hospital admission.

**Figure 1:**
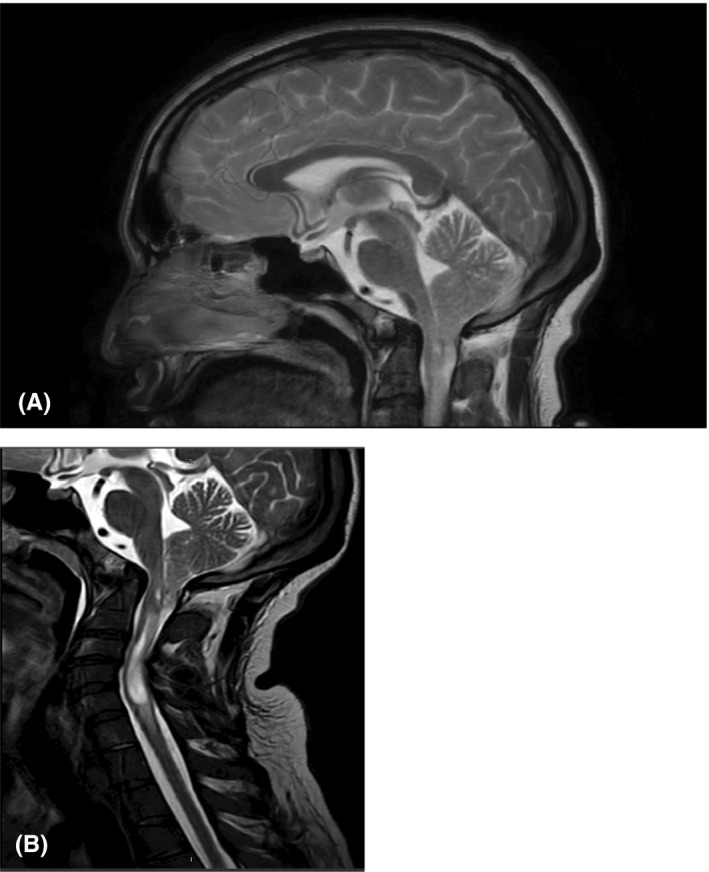
A and B, Sagittal magnetic resonance image of the brain. Note the cervico‐medullary junction compression

**Figure 2:**
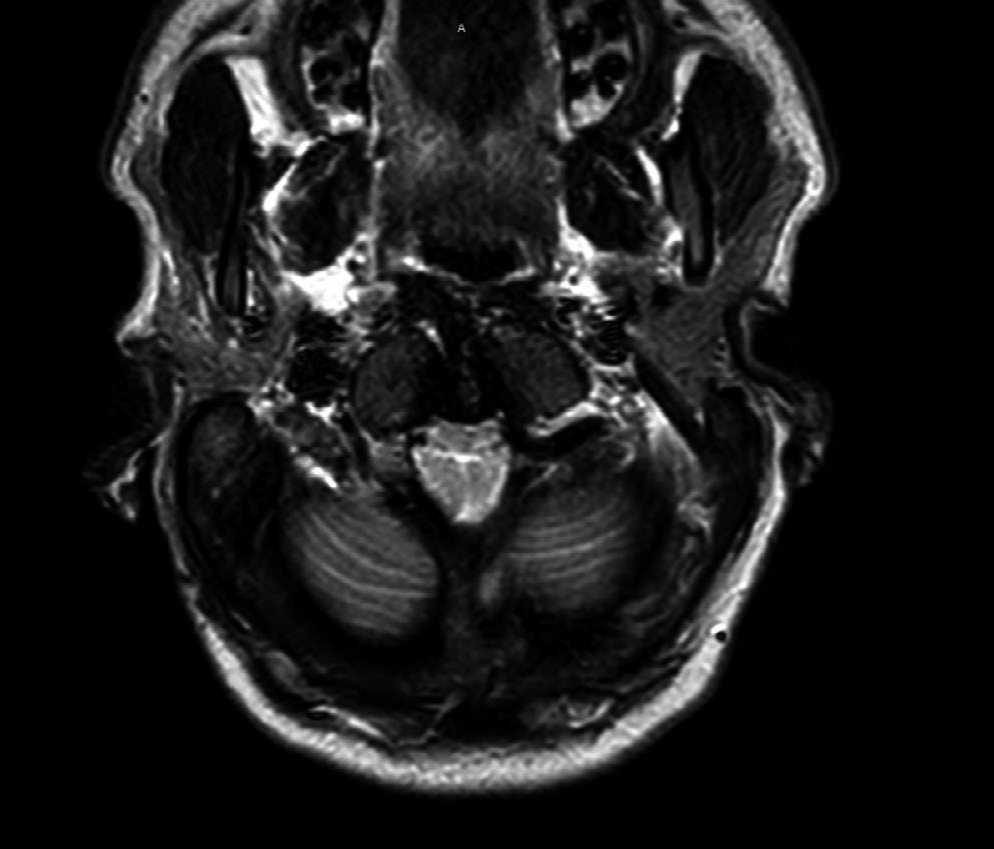
Axial C‐spine T2‐weighted magnetic resonance image of the upper cervical spinal cord at the level of C1‐2

In view of persistent type 2 respiratory failure which is unexplainable by pulmonary pathology and mild occasional difficulty of swallowing and bradypnea, neurological evaluation was done showed absent gag reflex, horizontal nystagmus, and wide base walking. Deep tendon reflexes ware exaggerated with impaired sensation in lower limbs. Thin liquid postswallowing cough was noted, pain while swallowing, so feeding nasogastric tube was inserted. MRI brain and cervical spine (see Figure 1 and Figure 2) was done, which revealed Arnold‐Chiari malformation with upper cervical syrinx suggestive of a type I ACM with associated cervico‐medullary junction compression, secondary causes were excluded. The patient was transferred to the neuro‐surgery unit for surgical decompression with cervical fixation (Glasgow Coma Score (GCS) was 15/15). Fixation has been done due to the craniocervical instability and reduction basilar invagination intraoperatively (fixation done to C5 because he has severe stenosis at level of C4/5 and cord signals and done for him C4/5 laminectomy). Postoperation, breathing, lower cranial nerve swallowing, gag reflex, coordination, and spasticity all were improved.

## 3 DISCUSSION

Respiration is regulated by two separate neural mechanism—voluntary and automatic.^7^ The automatic process is regulated by the respiratory centers in the brainstem. The chemoreceptors in the carotid and aortic bodies send signals to the medulla which innervates the respiratory muscles. In awake state, there is a continuous voluntary mechanism to maintain ventilation. During sleep, the voluntary mechanism is lost and anatomical and functional integrity become more important.^8^

Here, in this case, our patient had a healthy life with no prior history of any chronic respiratory illness until he developed a viral pneumonia. He developed acute type 2 respiratory failure requiring noninvasive ventilation. His arterial blood gas characteristics were suggestive of alveolar hypoventilation. His respiratory failure did not improve even after the treatment of pneumonia. Persistent unexplainable respiratory failure and bradypnea made us think of an alternate diagnosis of central cause for hypoventilation which was supported by his new onset occasional difficulty in swallowing. MRI substantiated our diagnosis. His hypoventilation was masked until he had a normal life. Central hypoventilation became evident when he had increased demand of ventilation due acute illness.

Type I is the most common form of ACM and has been estimated to occur in 1 in 1000 births and mostly asymptomatic. ACMs cases are often detected coincidently while undergoing unrelated investigations.^9^ ACM type I is no longer listed as a rare disorder by the Office of Rare Diseases Research (ORDR), as this classification is based on outdated data, mostly before the MRI era. The introduction of MRI has increased the frequency of ACM case discoveries.

The prevalence rate of type I ACM is ranged between 0.1%‐0.5% and less predominant in males. ^10^ Genetic basis for type I ACM has been often suggested.^11^, ^12^ Recent studies favor the presence of genetic linkage to chromosomes 9 and 15.^13^ Other researchers suggested that type I ACM is a disorder of primary para‐axial mesodermal origin, which could affect the formation of axial skeletal defect and different neurological anomalies.^14^ It can also be caused later in life if spinal fluid is drained excessively from the lumbar or thoracic areas of the spine either due to traumatic injury, disease, or infection. This is called acquired or secondary Chiari malformation. Primary Chiari malformation is much more common than secondary Chiari malformation.^15^

Type I ACM is a common structural disorder characterized by motor deficiency, sensory loss, lower cranial palsy, and cerebellar syndrome.^16^, ^17^, ^18^, ^19^ Neurological symptoms associated with Type I ACM are including mild to severe sensory deficit, lower cranial nerve palsies, headache, neck pain, and ataxia while respiratory‐related signs and symptoms seem to be unusual^20^ or properly unmasked by other conditions,^18^ and often associated with central alveolar hypoventilation^18^, ^20^ which is usually the trigger for clinicians to consider ACM not the respiratory failure, and this is typically what led us to make this observation. To the authors' knowledge, respiratory failure as the first manifestation of ACM has only been reported once in the English literature.^16^

## 4 CONCLUSIONS

In conclusion, ACM type I is no longer considered as a rare disease, and therefore, we urge clinicians to consider the possibility of ACM as well as other neurological causes in cases where acute respiratory failure is unexplainable.

## CONFLICT OF INTEREST

None declared.

## AUTHORS' CONTRIBUTIONS

MKA: Data Collection, Literature Search, Manuscript Preparation. MSE, AMS, DPM, DC, NK, NA, RMJ, AJN: Manuscript Preparation. All authors read and approved the final manuscript.

## ETHICS APPROVAL AND CONSENT TO PARTICIPATE

The article describes a case report. Therefore, no additional permission from our Ethics Committee was required.

## CONSENT FOR PUBLICATION

The consent for publication was obtained.

## Data Availability

All data generated during this study are included in this published article.
